# RKIP as an Inflammatory and Immune System Modulator: Implications in Cancer

**DOI:** 10.3390/biom9120769

**Published:** 2019-11-22

**Authors:** Maria Gabriela-Freitas, Joana Pinheiro, Ana Raquel-Cunha, Diana Cardoso-Carneiro, Olga Martinho

**Affiliations:** 1 Life and Health Sciences Research Institute (ICVS), School of Medicine, University of Minho, Campus de Gualtar, 4710-057 Braga, Portugal; pg36885@alunos.uminho.pt (M.G.-F.); pg36948@alunos.uminho.pt (J.P.); id8805@alunos.uminho.pt (A.R.-C.); id7787@alunos.uminho.pt (D.C.-C.); 2 ICVS/3Bs-PT Government Associate Laboratory, 4710-057 Braga/4805-017 Guimarães, Portugal; 3 Molecular Oncology Research Center, Barretos Cancer Hospital, Barretos, São Paulo 14784 400, Brazil

**Keywords:** RKIP, tumour microenvironment, inflammation, immunomodulation

## Abstract

Raf kinase inhibitor protein (RKIP), an important modulator of intracellular signalling pathways, is commonly downregulated in multiple cancers. This reduction, or loss of expression, is correlated not only with the presence of metastasis, contributing to RKIP’s classification as a metastasis suppressor, but also with tumour aggressiveness and poor prognosis. Recent findings suggest a strong involvement of RKIP in the modulation of tumour microenvironment components, particularly by controlling the infiltration of specific immune cells and secretion of pro-metastatic factors. Additionally, RKIP interaction with multiple signalling molecules seems to potentiate its function as a regulator of inflammatory processes, mainly through stimulation of anti- or pro-inflammatory cytokines. Furthermore, RKIP is involved in the modulation of immunotherapeutic drugs response, through diverse mechanisms that sensitize cells to apoptosis. In the present review, we will provide updated information about the role of RKIP as an inflammatory and immune modulator and its potential implications in cancer will be addressed.

## 1. Introduction

Raf kinase inhibitor protein (RKIP), also known as phosphatidylethanolamine-binding protein 1 (PEBP1), is a highly conserved, small (23 kDa), cytosolic protein originally purified from bovine brain [1,2]. This protein is widely expressed in normal human tissues, being recognized as having an important role in multiple physiological processes, such as spermatogenesis, neural development, cardiac output and membrane biosynthesis [2,3].

This multifunctional capacity of RKIP is associated with its involvement in the modulation of several signalling pathways (Reviewed at [4,5,6,7,8]). This protein was first described as a regulator of the Raf–MEK–ERK pathway, acting as its endogenous inhibitor. RKIP binds specifically to Raf-1 kinase, preventing its kinetic activity through the dissociation of the Raf-1/MEK complex, functioning as a competitive inhibitor of MEK phosphorylation [7,8,9]. Additionally, RKIP can indirectly interfere with upstream activators of Raf-1, such as G-protein coupled receptors (GPCR). Thus, when RKIP is phosphorylated by protein kinase C (PKC), it is released from Raf-1 and associates with G protein-coupled receptor kinase 2 (GRK2), an inhibitor of GPCR [10]. This association between phosphorylated RKIP and GRK2, not only leads to an enhanced GPCR activation, but also contributes to the overactivation of MAPK, since Raf-1 will no longer be inhibited by RKIP, ultimately leading to the activation of downstream targets. Therefore, RKIP will influence the cell’s response to growth factor stimuli [7]. Furthermore, RKIP can also act as a negative modulator of nuclear factor kappa B (NF-κB) signalling. This antagonizing effect of RKIP is exerted by its association with upstream kinases NIK, TAK, IKKα, and IKKβ, inhibiting their kinase activity, ultimately resulting in elimination of the IkappaB α (IκBα) phosphorylation and degradation, avoiding NF-κB translocation to the nucleus and consequently the transcription of several genes with anti-apoptotic features [11]. Moreover, RKIP also blocks signal transducer and activator of transcription 3 (STAT3) activation, by preventing its phosphorylation by upstream kinases, controlling the transcription of genes related to cell growth, apoptosis, survival and differentiation, [12,13]. Besides acting as an endogenous inhibitor in several signalling pathways, RKIP can also act as a positive modulator, as it is able to activate glycogen synthase kinase-3β (GSK3β) signalling, by preventing the phosphorylation of GSK3β inhibitory residue mediated by p38 MAPK and consequently stabilizing GSK3β expression [14].

Due to its important role as a modulator of intracellular signalling pathways that control several cellular processes, the deregulated expression of RKIP is implicated in several pathologies, including cancer [4,6]. The first association between RKIP and cancer was established in prostate metastatic cell lines, in which cellular RKIP expression levels were lower when compared to primary tumour cell lines [15]. It was also demonstrated that when RKIP expression was re-established in metastatic cells, their invasion capacity was inhibited, but the growth of the primary tumour was not affected [15]. This suggested that RKIP may not have a fundamental role in the primary tumour, but instead has great importance as a metastasis suppressor. In accordance, loss or reduction of RKIP expression is associated with malignancy and poor prognosis in several tumour types, as reported by our [16,17,18,19,20,21,22] and other groups [4,5,18,23,24,25,26]. Biologically, RKIP is a multifunctional protein in carcinogenesis, regulating cellular growth [27,28], motility [29,30], epithelial-to-mesenchymal transition (EMT) [31] and invasion [32]. Notably, it was also recognised that RKIP downregulation leads to inhibition of apoptosis and development of resistance to conventional cytotoxic drugs in tumour cells [5,33]. Furthermore, RKIP has an important role as a negative regulator of autophagy, by directly interfering with LC3-interaction region (LIR) motif, hampering autophagosome formation under starvation conditions [34]. Although the studies on this issue are scarce, they have hypothesized that RKIP’s regulation of cellular maintenance, chemo-immune resistance and EMT is driven by autophagy [35,36,37]. Interestingly, as recently reviewed by Wang et al. [35], RKIP and autophagy can both regulate the metastatic progression through EMT modulation, and curiously, they described that the RKIP/autophagy axis could be important to prevent oncogenic activation, cooperating to decrease cell cycle re-entry and re-establishment of genome stability [35].

Actually, one of the hot topics of research on cancer field is the belief that the tumor-immune system communications form the basis for disease pathophysiology and, at the same time, targets for therapeutic intervention [38,39]. The disease landscape emerging from these multi-factorial interactions is orchestrated by the three compartments, i.e., the cancer, the immune system, and the host. The outputs are numerous and include mainly: immunity that might control cancer and chronic inflammation that can be linked with tissue remodeling processes [40,41]. Inflammation is a hallmark of cancer and is mediated by immune cells attracted to or residing at sites of neoplastic transformation [42,43]. In what concerns the involvement of RKIP protein in the immuno-oncology topic, there is already some disperse information that strongly suggest a role for RKIP in the modulation of tumour microenvironment components, specifically in controlling the infiltration of specific immune cells and secretion of pro-metastatic factors [44,45]. Moreover, emerging data has been showing that RKIP, through interactions with numerous signalling molecules, may exert multiple functions during inflammatory processes [46]. RKIP is further reported to be involved in the modulation of the response to immunotherapeutic drugs and immuno-mediated cytotoxicity, by functioning as an apoptosis inducer, causing re-sensitization of resistant tumour cells and host immunosurveillance [47,48].

Hence, in the following sections we will provide an overview and updated information about RKIP function as an inflammatory and immune modulator and, accordingly, we will discuss its implications in oncological diseases.

## 2. RKIP and Inflammation

**Figure 1:**
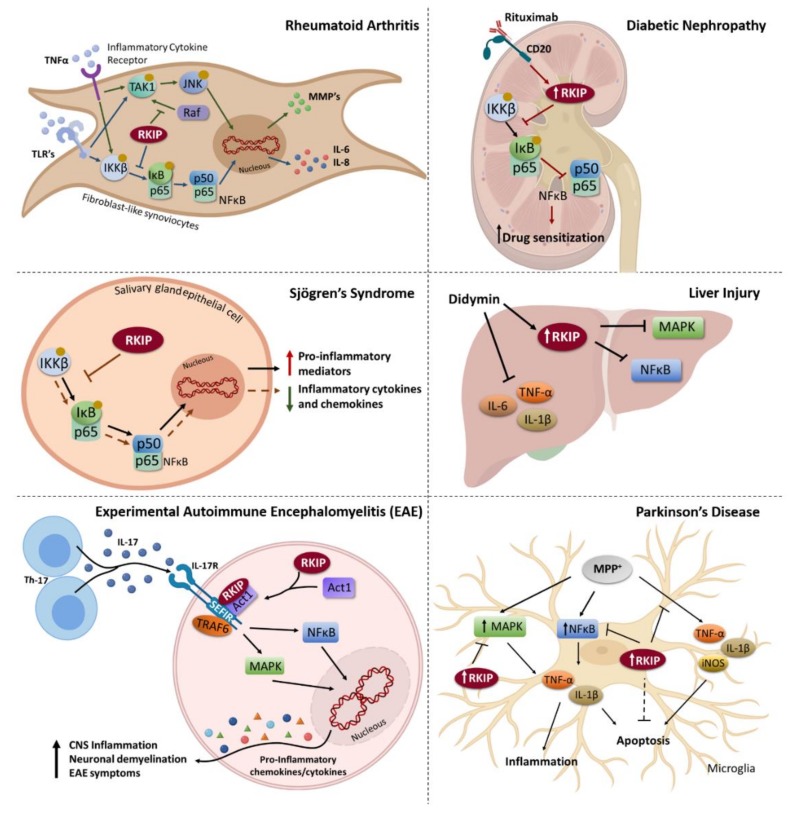
Raf kinase inhibitor protein (RKIP) as a modulator of signalling pathways involved in the regulation of inflammatory processes during different human pathologies. In rheumatoid arthritis, RKIP is constitutively expressed in fibroblast-like synoviocytes, being capable of decreasing the production of inflammatory cytokines and MMP’s through inhibition of ERK and NF-κB signalling. In diabetic nephropathy, Rituximab is able to upregulate RKIP, which in turn inhibits NF-κB pathway, leading to a drug sensitization. Likewise, in primary Sjögren’s syndrome (pSS), RKIP overexpression decreases NF-κB activity and subsequently contributes to the downregulation of inflammatory cytokines and chemokines, while RKIP silencing activates NF-κB signalling and increases the production of pro-inflammatory mediators. Didymin ameliorates liver injury through reduction of the levels of pro-inflammatory cytokines, and also by enhancing RKIP expression, thereby inhibiting MAPK and NF-κB signalling pathways. RKIP also plays a main role in Th17-mediated immune responses by positively regulating IL-17-induced pro-inflammatory cytokine and chemokine expression in experimental autoimmune encephalomyelitis (EAE). Specifically, RKIP promotes the formation of an IL-17R-Act1 complex, resulting in enhanced MAPK- and P65-mediated NF-κB activation and consequently triggering the production of pro-inflammatory cytokines and chemokines. This will ultimately promote disseminated CNS (central nervous system) inflammation, neuronal demyelination, and the development of EAE symptoms. In Parkinson’s disease, RKIP suppresses apoptosis and inflammation in MPP^+^-treated microglial cells through the inactivation of NF-κB and MEK/ERK signalling pathways.

Some studies have shown that RKIP could be a modulator of the inflammatory process through interaction with numerous signalling molecules that together regulate immune system response to inflammation mainly by the production of anti- or pro-inflammatory cytokines (Figure 1).

As mentioned before, RKIP is a known inhibitor of ERK/MAPK and NF-κB pathways, both of which are considered of great importance in inflammation [49,50]. Consequently, interactions of RKIP with them are likely to have an impact on ERK/MAPK and NF-κB-associated inflammatory processes. For instance, in rheumatoid arthritis, an NF-κB-associated chronic systemic inflammatory disease, overexpression of RKIP in fibroblast-like synoviocytes significantly decreases the expression of inflammatory cytokines (Figure 1). These effects are consistent with an enhanced ability of RKIP to inhibit NF-κB signalling [51]. Similarly, an interaction of RKIP with NF-κB has been suggested to occur in primary Sjögren’s syndrome (pSS), an autoimmune disorder characterized by an epithelial injury surrounded by dense lymphocytic infiltrates. The authors showed that upregulation of RKIP decreased NF-κB activity and contributed to the downregulation of inflammatory cytokines and chemokines (Figure 1). On the other hand, RKIP silencing significantly activated NF-κB signalling and increased expression and release of pro-inflammatory mediators. Interestingly, RKIP expression in salivary gland epithelial cells (SGEC) from pSS patients was reported to be significantly lower than that of healthy SGEC, supporting a role for RKIP in the suppression of NF-κB activation in pSS [52].

Additionally, it was described that the flavone didymin, isolated from *Origanum vulgare*, significantly ameliorated liver injury in mice by in part reducing the levels of pro-inflammatory cytokines, such as TNF-α, IL-6 and IL-1β (Figure 1). Didymin also enhanced RKIP expression, resulting in inhibition of the MAPK and NF-κB signalling pathways, which also contributed to its anti-inflammatory effect [53]. Furthermore, a study showed a decreased RKIP expression and increased NF-κB activation in renal tissues of rats with diabetic nephropathy (Figure 1), again supporting an inhibitory role for RKIP in NF-κB mediated inflammatory responses. Also, the authors proposed that Rituximab, a chimeric mouse anti-human CD20 monoclonal antibody, might promote RKIP expression as well, increasing the sensitivity of the renal tissues to this drug [54]. With the upregulation of RKIP, induced by Rituximab, NF-κB pathway is inhibited, hampering the pathological mechanism of diabetic nephropathy.

In addition, RKIP seems to have different roles in the modulation of several inflammatory diseases. For example, in lung inflammation, RKIP is capable of regulating the cell type and signalling specific expression of the enzyme protein arginine methyltransferase 1 (PRMT1). More precisely, RKIP was found to be highly expressed in epithelial cells, preventing IL-1β-induced stimulation of PRMT1 expression [55]. RKIP also mediates autoimmune inflammation, since its deficiency in mice ameliorates the symptoms of experimental autoimmune encephalomyelitis (EAE), an experimental model for multiple sclerosis (Figure 1). Specifically, it was found that RKIP plays a main role in Th17-mediated immune responses by positively regulating IL-17-induced pro-inflammatory cytokine and chemokine expression. RKIP seems to directly interact with IL-17R and Act1 to promote the formation of an IL-17R-Act1 complex, resulting in enhanced MAPK- and P65-mediated NF-κB activation and downstream cytokine production (Figure 1) [56]. Moreover, RKIP plays an important role in controlling mast cell mediated allergic responses, specifically by negatively regulating mast cell activation. It was described that RKIP-deficient mast cells showed greater activation than wild-type mast cells and, consistently, RKIP deficiency in mast cells rendered mice more sensitive to allergic responses and ovalbumin-induced airway inflammation. Mechanistically, RKIP interacts with PI3K, preventing it from binding to GRB2-associated binding protein 2 (Gab2), and eventually inhibiting the activation of the PI3K/AKT/NF-κB complex and its downstream signalling [57].

Focusing on the regulation of neural development, the role of RKIP in microglial cells stimulated with 1-methyl-4-phenylpyridinium (MPP^+^) and its underlying mechanism in Parkinson’s disease was investigated. The findings indicated that RKIP suppresses apoptosis and inflammation in MPP^+^-treated microglial cells through the inactivation of NF-κB and MEK/ERK signalling pathways (Figure 1) [58]. Additionally, a study by Wang et al. reported that RKIP expression was dramatically reduced by erythrocyte lysate treatment in microglia, and restoration of RKIP distinctly inhibited microglia inflammation through inhibition of the NF-κB signalling pathway in erythrocyte lysis-treated microglial cells [59].

**Figure 2:**
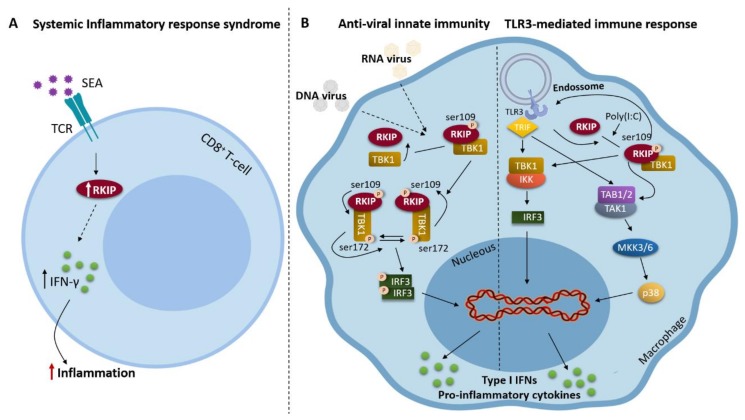
Role of RKIP in interferons and pro-inflammatory cytokines production during inflammatory processes. (**A**) RKIP enhances the production of interferon-γ after triggering of the T cell receptor (TCR) with staphylococcal enterotoxin A (SEA) in CD8^+^ systemic inflammatory response syndrome (SIRS) T cells. (**B**) On the left side, upon viral infection, TBK1 phosphorylates RKIP at S109 in macrophages. The phosphorylation of RKIP enhances its interaction with TBK1 and in turn promotes TBK1 autophosphorylation at S172, thus triggering a positive feedback control of TBK1 activation, which is essential for type I interferon production in innate immunity. On the right side, RKIP is a positive regulator of the TLR-3-mediated immune response in macrophages, being required for TBK1-IRF3 and MKK3-p38 activation and the downstream production of type I interferon and proinflammatory cytokines. TLR3 activation induces phosphorylation of RKIP at S109 via TBK1. Phosphorylated RKIP promotes TBK1 activation and the interaction between TAK1 and MKK3, thus activating the downstream IRF3 and p38, respectively.

RKIP also has a particularly important role in driving the production of type I and type II interferons. In fact, RKIP showed to be critical for enhancing type II interferon production in CD8^+^ systemic inflammatory response syndrome (SIRS) T cells after serial triggering of the T cell receptor (TCR) with staphylococcal enterotoxin A (SEA) (Figure 2A). On a molecular level, the authors showed that this effect was not due to differences in T cell expansion or production of IL-10, an anti-inflammatory cytokine, but instead RKIP plays a role in the signalling machinery downstream of the TCR [60]. RKIP is also an important positive regulator of TANK-binding kinase 1 (TBK1) activation and type I interferon production in innate immunity. Upon viral infection, RKIP is phosphorylated at serine 109 (S109) by TBK1, which enhances the interaction between these two molecules and in turn promotes TBK1 autophosphorylation (S172). In contrast, RKIP deficiency inhibits intracellular double-stranded RNA- or DNA-induced type I interferon production (Figure 2B). These findings revealed a positive feedback loop between RKIP and TBK1 that is essential for type I interferon production in anti-viral innate immunity [61]. Besides, RKIP preferentially positively regulates the TLR3-mediated immune response in macrophages. It was shown that RKIP deficiency remarkably impaired polyinosinic:polycytidylic acid-induced TBK1/IRF3 and MAPK kinase 3 (MKK3)/p38 activation, and significantly inhibited Poly(I:C)-induced pro-inflammatory cytokine production in macrophages (Figure 2B). In addition, RKIP-deficient mice produced fewer pro-inflammatory cytokines and type I interferons and were more resistant to Poly(I:C)-induced death. It was further demonstrated that Poly(I:C) treatment induces RKIP phosphorylation at S109, being this action required for RKIP to promote TLR3-mediated signalling and inflammation [62].

Recently, it was demonstrated that RKIP also plays a relevant role in mediating human and mouse colitis by promoting inflammation and mediating intestinal epithelial cell apoptosis. The results showed that RKIP deficiency protects from colitis and inhibits infiltration of acute-phase immune cells and reduces production of pro-inflammatory cytokines and chemokines [63]. Mechanistically, RKIP enhances the induction of P53-upregulated modulator of apoptosis by interacting with TGF-β-activated kinase 1 (TAK1) and promoting TAK1-mediated NF-κB activation. This is supported by the observation that TAK1 activation is positively correlated with the expression of RKIP in human clinical samples [64]. Furthermore, a study showed that RKIP could be important in the regulation of Na-dependent amino acid absorption during chronic intestinal inflammation. The authors show that leukotriene D_4_ (LTD_4_) inhibits Na-alanine cotransport in intestinal epithelial cells by decreasing the affinity of the cotransporter ASCT1 by PKC-mediated phosphorylation of RKIP and leading to an activation of protein kinase A (PKA) pathway [63].

Altogether, these findings highlight the importance of RKIP’s role in inflammatory processes and its potential clinical applicability in therapeutic approaches for inflammatory diseases. In a study, inhibition of RKIP using the small molecule inhibitor locostatin led to a significantly diminished IFN-γ response in SIRS [60]. Locostatin alkylates a histidine residue (His68), a highly conserved residue in RKIP’s ligand-binding pocket, preventing RKIP from binding to its ligands and inhibiting RKIP functions [65]. Thus, this inhibitory effect of locostatin on RKIP associated with a decrease in IFN-γ suggested the potential of RKIP as a therapeutic target in inflammatory diseases.

Regarding cancer, inflammation has shown to be a critical component of tumour progression, fostering proliferation survival and migration through the recruitment of inflammatory and/or immune cells [66]. Little is known about the link between RKIP and inflammation in neoplastic context, however, given RKIP’s role in inflammatory processes through the modulation of signalling pathways, such as ERK/MAPK and NF-κB pathways, that are known to be widely deregulated in several malignancies (Figure 1 and Figure 2), it is possible that RKIP’s role in cancer can also be mediated by inflammation. It was already reported in breast and prostate cancer cell lines, that IL-6-mediated STAT3 activation showed to be RKIP-dependent as it was demonstrated by the lack of STAT3 activation in a human breast carcinoma cell line overexpressing RKIP and a significant enhancement of activation in cells with RKIP knockdown [12]. Additionally, RKIP was found to interact with tumour necrosis factor receptor 19 (TROY) in glio ma cell lines, an interaction that was enhanced by fetal bovine serum (FBS) exposure. Disruption of the TROY/RKIP interaction reduced the glioma development in xenografted mice [67]. Altogether, such findings suggest that RKIP may potentially exert its tumour suppressor function through the modulation of inflammatory factors.

In summary, it is evident that RKIP has an important role in inflammation, either by regulating important signalling pathways, such as ERK/MAPK and NF-κB pathways, or by regulating pro-inflammatory cytokine production. However, much remains to be understood regarding the mechanisms by which RKIP regulates inflammation in neoplastic context. Hence, further investigation in this area is needed for the development of new therapeutic approaches targeting RKIP inflammatory function.

## 3. RKIP and Tumour Microenvironment

Recent findings suggest that RKIP’s anti-metastatic properties can also be mediated through the modulation of components of tumour microenvironment. The interaction between metastasis suppressors and components of the microenvironment has been evident in recent years. However, little is known about how the metastasis suppressors regulate the tumour microenvironment, and the importance of this regulation to metastasis suppression [44,68]. The tumour microenvironment consists of different cell populations, besides cancer and cancer stem cells, that collectively control and support tumour progression and metastasis. Among them are infiltrating immune cells, such as tumour-associated macrophages (TAMs) and myeloid cells, that are known to add a more invasive and pro-tumoural phenotype to tumour cells by secreting angiogenic and growth factors [69].

Within tumour microenvironment, cancer stem cells (CSCs) are tumour cells that have self-renewal properties, clonal tumour initiation capacity, and clonal long-term repopulation potential [70]. A recent study suggested that RKIP expression levels may be involved in the regulation of the cancer stem cell phenotype. In this latter study, the existence of a crosstalk in the signalling pathways between RKIP and several cancer stem cell transcription factors, namely Oct4, KLF4, Sox2 and Nanog, was assembled. The findings revealed that there is a direct correlation between RKIP expression and the expression of each of the above transcription factors, however this needs to be experimentally validated [71]. Moreover, Yang et al., comparing the expression of cluster of differentiation 44 (CD44), a well-studied tumour marker associated with gastric cancer stem cells, with RKIP expression under different pathological conditions, demonstrated that RKIP exhibited a negative effect on initial tumour development, and that the downregulation of RKIP in the advanced stages of cancer facilitated CD44 and peroxiredoxin 2 overexpression. These observations suggest that RKIP may play a role in gastric cancer stem cell expansion [72].

The first evidences of RKIP role in the modulation of tumour microenvironment were reported in 2015 by two groups, that separately demonstrated that RKIP controls TAMs’ infiltration in the breast cancer microenvironment, both in vitro and in vivo [44,45]. Frankenberger et al. demonstrated that RKIP expression in metastatic triple-negative breast tumours markedly reduces the number and metastatic potential of infiltrating TAMs, which by itself translated into a decrease in tumour cell invasiveness and secretion of pro-metastatic factors, namely progranulin (PRGN) and tumour necrosis factor receptor 2 (TNFR2). The underlying mechanism through which RKIP regulates TAM recruitment is an RKIP-mediated HMGA2 blockage, that in turn results in a reduced expression of numerous macrophage chemotactic factors, such as chemokine ligand 5 (CCL5) [44]. Almost simultaneously, Datar et al. demonstrated, using an orthotopic breast cancer model, that ectopic expression of RKIP significantly decreased tumour vasculature, macrophage infiltration and lung metastases, by inhibiting the expression of CCL5. These results were in accordance with the in vitro analysis that showed that RKIP hinders breast cancer cell invasion by inhibiting expression of the chemokine CCL5. Beyond this, they established for the first time an inverse correlation between RKIP and CCL5 expression levels in clinical human breast cancer samples [45]. Taken together, both studies identified the significance of RKIP as an important novel negative regulator of tumour microenvironment, at least by blocking the recruitment of pro-metastatic macrophages, through regulation of chemokines expression.

Furthermore, in a broader study, Bainer et al. demonstrated, using species-specific RNA sequencing in a xenograft TNBC (Triple-negative breast cancer) mouse model, that gene expression in metastatic breast tumours is largely correlated with gene expression in local stroma of both mouse xenografts and human patients. In addition, changes in stromal gene expression elicited by tumours with or without RKIP expression is a better predictor of breast cancer subtype and patient survival than tumour gene expression [73]. In a recent study, Buschow et al. revealed that RKIP expression, during immunotherapy of metastatic melanoma with dendritic cell (DC) vaccination, positively correlated with gene signatures involved in effective T cell responses, but inversely correlated with genes associated with myeloid cell infiltration and inflammation, such as STAT3, Notch1, and MAPK1 signalling members [74]. Besides that, RKIP inversely correlated with the myeloid/lymphoid-ratio and was suppressed in patients suffering from chronic inflammatory disease, suggesting that RKIP may be indicative of a skewing of the (DC-vaccine-triggered) immune response towards chronic inflammation/myeloid immune suppression rather than towards an effective anti-tumour response [74]. Similarly, it was demonstrated in gastric cardiac adenocarcinoma tissues that negative RKIP expression was correlated with lower T cell-mediated immune function in the tumour microenvironment and increased lymph node metastasis, possibly by a mechanism of NF-κB hyperactivity [75]. Moreover, in chronic lymphocytic leukemia (CLL), inhibition of RKIP by locostatin led to a decreased expression of the chemokine receptor CXCR4 and reduced the migratory capacity of CLL cells toward stroma-derived factor 1a (SDF-1a), being these effects of locostatin possibly mediated by the binding of GRK2 to MEK1 and AKT [76]. Altogether, these observations highlight the importance of exploiting RKIP’s microenvironmental functions in order to develop novel therapeutic approaches for cancer patients.

## 4. RKIP and Cancer Immunomodulation

Tumour cells frequently develop therapeutic resistance, resulting in a poorer overall survival. In many cases, therapeutic resistance is concomitant with increased capacity of tumour cells escaping to host immunosurveillance. One approach to overcome this problem has been to identify pathways that regulate resistance and develop means to disrupt these pathways in order to re-sensitize resistant cells to death.

**Figure 3:**
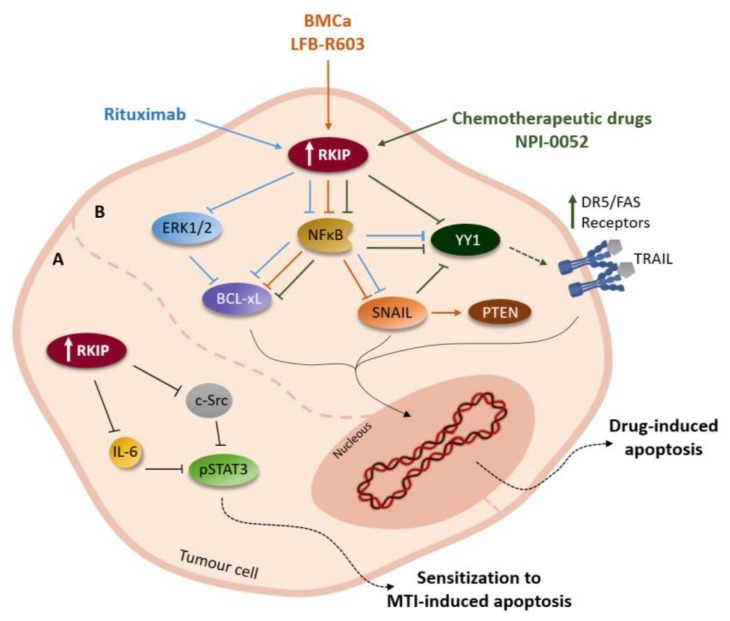
RKIP-mediated signalling accountable for drug-induced apoptosis sensitization in cancer cells. (**A**) Overexpression of RKIP leads to reduction of c-Src- and IL-6 -mediated signal transducer and activator of transcription 3 (STAT3) activation. Such will ultimately result in cell sensitization to Microtubule Inhibitors (MTI)-induced apoptosis. (**B**) Treatment of tumour cells with therapies such as chemotherapeutic drugs and monoclonal antibodies have as mechanism of action the upregulation of RKIP. This will result in the inhibition of pathways such as NF-κB and MAPK, which in turn will block proteins like Bcl-xL and transcription factors like Yin Yang 1 (YY1) and Snail, culminating in upregulation of death receptor 5 (DR5) expression and derepression of phosphatase and tensin homolog (PTEN), respectively. These events sensitize cells to drug-induced apoptosis, including through TNF-related apoptosis-inducing ligand (TRAIL)-induced apoptosis. Each colour represents the different groups of drugs.

In that sense, RKIP has been identified as an important modulator of tumour cells therapy response via multiple interactions with signalling modules [33]. As an example, in the Yousuf et al. study, it was reported that RKIP overexpression results in the reduction of STAT3 activation mediated by IL-6 and c-Src, which resulted in sensitization of the cells to microtubule inhibitors (MTI)-induced apoptosis (Figure 3) [12]. In fact, some agents have already been reported as able to induct RKIP expression, resulting in the reversal of resistance and sensitization to TNF-related apoptosis-inducing ligand (TRAIL) and Fas ligand (Fas-L)-mediated apoptosis, two important mechanisms for tumour cells death by the immune system [47,48,77,78,79]. Baritaki et al. reported that treatment of tumour cells with various chemotherapeutic drugs not only inhibits NF-κB activity but also sensitizes the cells to TRAIL-induced apoptosis concurrently with upregulation of death receptor 5 (DR5) expression and inhibition of the transcription repressor Yin Yang 1 (YY1) [77]. Interestingly, a different study showed that RKIP induction resulted in the inhibition of YY1 and sensitization to TRAIL-mediated apoptosis alongside with upregulation of DR5, while treatment of tumour cells with RKIP small interfering RNA (siRNA) reversed tumour cell sensitization to TRAIL [48]. These results suggest that the underlying mechanism of RKIP-induced sensitization to TRAIL is inhibition of NF-κB and YY1 and augmentation of DR5 expression by RKIP (Figure 3).

Furthermore, the proteasome inhibitor NPI-0052 showed to sensitize cells to TRAIL-mediated apoptosis by inhibiting both NF-κB and Snail and inducing RKIP expression. The authors corroborated the direct role of NF-κB inhibition in sensitization by treatment with DHMEQ (Dehydroxymethylepoxyquinomicin), a NF-κB inhibitor, which mimicked NPI-0052 in the inhibition of NF-κB and Snail, along with upregulation of RKIP and sensitization to TRAIL (Figure 3). Also, treatment with Snail siRNA reversed resistance and induced RKIP overexpression. Likewise, RKIP overexpression mimicked treatment with Snail siRNA or NPI-0052 in sensitization of cells to TRAIL apoptosis, concomitant with suppression of the anti-apoptotic gene Bcl-xL (Figure 3). In contrast, treatment with RKIP siRNA reversed the sensitization to TRAIL [80].

Collectively, these findings established that NF-κB/Snail/YY1/RKIP circuitry regulates tumour cell sensitivity to TRAIL-mediated apoptosis. Nevertheless, RKIP-induced inhibition of the Raf/MEK/ERK pathway may also be involved in sensitization, since this pathway was reported to abrogate the apoptotic signalling by death receptors, including TRAIL [81]. Thus, there is evidence that RKIP may serve as an immune surveillance cancer gene, and its low expression or absence in tumours allows them to escape host immune cytotoxic cells.

Importantly, RKIP demonstrated a significant role in the modulation of immunomodulatory drugs response, through diverse mechanisms that sensitize cells to apoptosis. Some antibodies, such as the immunomodulators Rituximab and LFB-R603, have been reported as capable of sensitizing cells to apoptosis through mechanisms involving RKIP upregulation [47,82,83]. It was described that Rituximab sensitizes non-Hodgkin’s lymphoma B cells to paclitaxel-induced apoptosis, due to its ability to interrupt the ERK1/2 signalling pathway, which is concomitant with Bcl-xL downregulation (Figure 3). Looking further into the molecular mechanism, the authors observed that rituximab inhibits ERK1/2 pathway, not only through the upregulation of RKIP, but also through the potentiation of RKIP/Raf-1 association [47,83]. Moreover, Rituximab-mediated chemosensitization could also be due to inhibition of the constitutive NF-κB pathway, which is partly responsible for the regulation of Bcl-xL expression. Rituximab upregulates RKIP expression, potentiating the association between RKIP and the inhibitory NF-κB upstream kinases [47,84].

Similarly, two other monoclonal antibodies targeting CD-20, LFB-R603 and BM-ca, showed the same results [82,85]. LFB-R603 is able to sensitize non-Hodgkin’s lymphoma B cells to apoptosis mediated by TRAIL. The underlying mechanism of sensitization is an inhibitory effect of LFB-R603 on the constitutively activated NF-κB and PI3K/AKT survival pathways. LFB-R603-mediated NF-κB inhibition results in the downstream inhibition of Snail, concomitantly with the derepression of RKIP and phosphatase and tensin homolog (PTEN). Given that PTEN is an endogenous inhibitor of PI3K/AKT, suppressed by Snail, it was suggested that RKIP-mediated inhibition of NF-κB/Snail signalling may cause PI3K/AKT suppression in consequence of PTEN induction. Hence, LFB-R603 is responsible for the sensitization to TRAIL-mediated apoptosis through the regulation of NF-κB/Snail/RKIP/PTEN circuitry [82]. Concerning BM-ca, it was reported that following treatment with this antibody, the P38 MAPK and NF-κB pathways are inhibited, resulting in the inhibition of Snail transcription and derepression of RKIP (Figure 3). Derepression of RKIP, in turn, accentuates the inhibition of NF-κB and Snail, manifested by the inhibition of anti-apoptotic gene products and induction of pro-apoptotic gene products, leading to sensitization to drug-induced apoptosis [85].

Summarizing, there is accumulating evidence supporting a new role for RKIP in the regulation of the immune response, in addition to its previously identified metastatic suppressor role. Since the levels of RKIP are low in most tumours, its induction may inhibit metastasis and sensitize cells to both chemotherapeutic and immunomodulatory drugs.

## 5. Conclusions

RKIP is a multifunctional protein that is involved in many physiological processes, being considered a well-established metastasis suppressor in several tumour types. Despite the well-defined roles for RKIP in cancer, its function within the immune system is little understood. In this review, the relationship between RKIP and the immune system was elucidated, and it was possible to establish that RKIP has a role in the modulation of components of the tumour microenvironment, mainly by controlling the infiltration of specific immune cells and secretion of pro-metastatic factors. Besides that, RKIP demonstrated to have an important role in inflammation, mainly through interactions with several signalling molecules and modulation of cytokines production, both in inflammatory pathologies and cancer. In addition, RKIP showed to be involved in the modulation of several immunomodulatory drugs, mainly by mechanism of cells sensitization to apoptosis. In conclusion, the role of RKIP as an immunomodulator is evident, however a better understanding of its function in the immune system and its implications in the neoplastic context can be relevant for the development of therapies targeting RKIP function in tumour microenvironment, inflammation and immunosurveillance.

## References

[1] Al-Mulla F., Bitar M.S., Taqi Z., Yeung K.C. (2013). RKIP: Much more than Raf kinase inhibitory protein. J. Cell Physiol..

[2] Keller E.T., Fu Z., Brennan M. (2004). The role of Raf kinase inhibitor protein (RKIP) in health and disease. Biochem. Pharmacol..

[3] Klysik J., Theroux S.J., Sedivy J.M., Moffit J.S., Boekelheide K. (2008). Signaling crossroads: The function of Raf kinase inhibitory protein in cancer, the central nervous system and reproduction. Cell. Signal..

[4] Yesilkanal A.E., Rosner M.R. (2018). Targeting Raf Kinase Inhibitory Protein Regulation and Function. Cancers.

[5] Zaravinos A., Bonavida B., Chatzaki E., Baritaki S. (2018). RKIP: A Key Regulator in Tumor Metastasis Initiation and Resistance to Apoptosis: Therapeutic Targeting and Impact. Cancers.

[6] Farooqi A.A., Li Y., Sarkar F.H. (2015). The biological complexity of RKIP signaling in human cancers. Exp. Mol. Med..

[7] Yesilkanal A.E., Rosner M.R. (2014). Raf kinase inhibitory protein (RKIP) as a metastasis suppressor: Regulation of signaling networks in cancer. Crit. Rev. Oncog..

[8] Vandamme D., Herrero A., Al-Mulla F., Kolch W. (2014). Regulation of the MAPK pathway by raf kinase inhibitory protein. Crit. Rev. Oncog..

[9] Yeung K., Janosch P., McFerran B., Rose D.W., Mischak H., Sedivy J.M., Kolch W. (2000). Mechanism of suppression of the Raf/MEK/extracellular signal-regulated kinase pathway by the raf kinase inhibitor protein. Mol. Cell. Biol..

[10] Lorenz K., Lohse M.J., Quitterer U. (2003). Protein kinase C switches the Raf kinase inhibitor from Raf-1 to GRK-2. Nature.

[11] Yeung K.C., Rose D.W., Dhillon A.S., Yaros D., Gustafsson M., Chatterjee D., McFerran B., Wyche J., Kolch W., Sedivy J.M. (2001). Raf kinase inhibitor protein interacts with NF-kappaB-inducing kinase and TAK1 and inhibits NF-kappaB activation. Mol. Cell. Biol..

[12] Yousuf S., Duan M., Moen E.L., Cross-Knorr S., Brilliant K., Bonavida B., LaValle T., Yeung K.C., Al-Mulla F., Chin E. (2014). Raf kinase inhibitor protein (RKIP) blocks signal transducer and activator of transcription 3 (STAT3) activation in breast and prostate cancer. PLoS ONE.

[13] Wang A., Duan G., Zhao C., Gao Y., Liu X., Wang Z., Li W., Wang K., Wang W. (2017). Reduced RKIP expression levels are associated with frequent non-small cell lung cancer metastasis and STAT3 phosphorylation and activation. Oncol. Lett..

[14] Al-Mulla F., Bitar M.S., Al-Maghrebi M., Behbehani A.I., Al-Ali W., Rath O., Doyle B., Tan K.Y., Pitt A., Kolch W. (2011). Raf kinase inhibitor protein RKIP enhances signaling by glycogen synthase kinase-3beta. Cancer Res..

[15] Fu Z., Smith P.C., Zhang L., Rubin M.A., Dunn R.L., Yao Z., Keller E.T. (2003). Effects of raf kinase inhibitor protein expression on suppression of prostate cancer metastasis. J. Natl. Cancer Inst..

[16] Martinho O., Granja S., Jaraquemada T., Caeiro C., Miranda-Goncalves V., Honavar M., Costa P., Damasceno M., Rosner M.R., Lopes J.M. (2012). Downregulation of RKIP is associated with poor outcome and malignant progression in gliomas. PLoS ONE.

[17] Martinho O., Faloppa C.C., Neto C.S., Longatto-Filho A., Baiocchi G., da Cunha I.W., Soares F.A., Fregnani J.H., Reis R.M. (2012). Loss of RKIP expression during the carcinogenic evolution of endometrial cancer. J. Clin. Pathol..

[18] Martinho O., Gouveia A., Silva P., Pimenta A., Reis R.M., Lopes J.M. (2009). Loss of RKIP expression is associated with poor survival in GISTs. Virchows Arch..

[19] Afonso J., Longatto-Filho A., Martinho O., Lobo F., Amaro T., Reis R.M., Santos L.L. (2013). Low RKIP expression associates with poor prognosis in bladder cancer patients. Virchows Arch. Int. J. Pathol..

[20] Martinho O., Pinto F., Granja S., Miranda-Goncalves V., Moreira M.A., Ribeiro L.F., di Loreto C., Rosner M.R., Longatto-Filho A., Reis R.M. (2013). RKIP inhibition in cervical cancer is associated with higher tumor aggressive behavior and resistance to cisplatin therapy. PLoS ONE.

[21] Martinho O., Campos M., Ribeiro G., Penna V., Curcelli E.C., Olivieri M.V., Morini S., Scapulatempo C., Abrahao-Machado L.F., Reis R.M. (2016). Raf Kinase Inhibitor Protein Expression and Prognostic Value in Soft Tissue Sarcomas. Pathobiology.

[22] Raquel-Cunha A., Cardoso-Carneiro D., Reis R.M., Martinho O. (2019). Current Status of Raf Kinase Inhibitor Protein (RKIP) in Lung Cancer: Behind RTK Signaling. Cells.

[23] Lamiman K., Keller J.M., Mizokami A., Zhang J., Keller E.T. (2014). Survey of Raf kinase inhibitor protein (RKIP) in multiple cancer types. Crit. Rev. Oncog..

[24] Kim H.S., Won K.Y., Kim G.Y., Kim S.C., Park Y.K., Kim Y.W. (2012). Reduced expression of Raf-1 kinase inhibitory protein predicts regional lymph node metastasis and shorter survival in esophageal squamous cell carcinoma. Pathol. Res. Pract..

[25] Wang Y., Wang L.Y., Feng F., Zhao Y., Huang M.Y., Shao Q., Chen C., Sheng H., Chen D.L., Zeng Z.L. (2015). Effect of Raf kinase inhibitor protein expression on malignant biological behavior and progression of colorectal cancer. Oncol. Rep..

[26] Martinho O., Simoes K., Longatto-Filho A., Jacob C.E., Zilberstein B., Bresciani C., Gama-Rodrigues J., Cecconello I., Alves V., Reis R.M. (2013). Absence of RKIP expression is an independent prognostic biomarker for gastric cancer patients. Oncol. Rep..

[27] Akaishi J., Onda M., Asaka S., Okamoto J., Miyamoto S., Nagahama M., Ito K., Kawanami O., Shimizu K. (2006). Growth-suppressive function of phosphatidylethanolamine-binding protein in anaplastic thyroid cancer. Anticancer Res..

[28] Zhang L., Fu Z., Binkley C., Giordano T., Burant C.F., Logsdon C.D., Simeone D.M. (2004). Raf kinase inhibitory protein inhibits beta-cell proliferation. Surgery.

[29] Bement W.M. (2005). A role for RKIP in cell motility. Chem. Biol..

[30] Al-Mulla F., Bitar M.S., Taqi Z., Rath O., Kolch W. (2011). RAF kinase inhibitory protein (RKIP) modulates cell cycle kinetics and motility. Mol. Biosyst..

[31] Baritaki S., Chapman A., Yeung K., Spandidos D.A., Palladino M., Bonavida B. (2009). Inhibition of epithelial to mesenchymal transition in metastatic prostate cancer cells by the novel proteasome inhibitor, NPI-0052: Pivotal roles of Snail repression and RKIP induction. Oncogene.

[32] Hellmann J., Rommelspacher H., Muhlbauer E., Wernicke C. (2010). Raf kinase inhibitor protein enhances neuronal differentiation in human SH-SY5Y cells. Dev. Neurosci..

[33] Al-Mulla F., Bitar M.S., Feng J., Park S., Yeung K.C. (2012). A new model for raf kinase inhibitory protein induced chemotherapeutic resistance. PLoS ONE.

[34] Noh H.S., Hah Y.S., Zada S., Ha J.H., Sim G., Hwang J.S., Lai T.H., Nguyen H.Q., Park J.Y., Kim H.J. (2016). PEBP1, a RAF kinase inhibitory protein, negatively regulates starvation-induced autophagy by direct interaction with LC3. Autophagy.

[35] Wang Y., Bonavida B. (2018). A New Linkage between the Tumor Suppressor RKIP and Autophagy: Targeted Therapeutics. Crit. Rev. Oncog..

[36] Ahmed M., Lai T.H., Zada S., Hwang J.S., Pham T.M., Yun M., Kim D.R. (2018). Functional Linkage of RKIP to the Epithelial to Mesenchymal Transition and Autophagy during the Development of Prostate Cancer. Cancers.

[37] Bonavida B. (2018). Linking Autophagy and the Dysregulated NFkappaB/SNAIL/YY1/RKIP/PTEN Loop in Cancer: Therapeutic Implications. Crit. Rev. Oncog..

[38] Demaria O., Cornen S., Daeron M., Morel Y., Medzhitov R., Vivier E. (2019). Harnessing innate immunity in cancer therapy. Nature.

[39] Shaked Y. (2019). The pro-tumorigenic host response to cancer therapies. Nat. Rev. Cancer.

[40] Palucka A.K., Coussens L.M. (2016). The Basis of Oncoimmunology. Cell.

[41] Roumenina L.T., Daugan M.V., Petitprez F. (2019). Context-dependent roles of complement in cancer. Nat. Rev. Cancer.

[42] Elinav E., Nowarski R., Thaiss C.A., Hu B., Jin C., Flavell R.A. (2013). Inflammation-induced cancer: Crosstalk between tumours, immune cells and microorganisms. Nat. Rev. Cancer.

[43] Balkwill F., Charles K.A., Mantovani A. (2005). Smoldering and polarized inflammation in the initiation and promotion of malignant disease. Cancer Cell.

[44] Frankenberger C., Rabe D., Bainer R., Sankarasharma D., Chada K., Krausz T., Gilad Y., Becker L., Rosner M.R. (2015). Metastasis Suppressors Regulate the Tumor Microenvironment by Blocking Recruitment of Prometastatic Tumor-Associated Macrophages. Cancer Res..

[45] Datar I., Qiu X., Ma H.Z., Yeung M., Aras S., de la Serna I., Al-Mulla F., Thiery J.P., Trumbly R., Fan X. (2015). RKIP regulates CCL5 expression to inhibit breast cancer invasion and metastasis by controlling macrophage infiltration. Oncotarget.

[46] Zhao J., Wenzel S. (2014). Interactions of RKIP with inflammatory signaling pathways. Crit. Rev. Oncog..

[47] Jazirehi A.R., Bonavida B. (2005). Cellular and molecular signal transduction pathways modulated by rituximab (rituxan, anti-CD20 mAb) in non-Hodgkin’s lymphoma: Implications in chemosensitization and therapeutic intervention. Oncogene.

[48] Baritaki S., Katsman A., Chatterjee D., Yeung K.C., Spandidos D.A., Bonavida B. (2007). Regulation of tumor cell sensitivity to TRAIL-induced apoptosis by the metastatic suppressor Raf kinase inhibitor protein via Yin Yang 1 inhibition and death receptor 5 up-regulation. J. Immunol..

[49] Kaminska B. (2005). MAPK signalling pathways as molecular targets for anti-inflammatory therapy--from molecular mechanisms to therapeutic benefits. Biochim. Biophys. Acta.

[50] Liu T., Zhang L., Joo D., Sun S.C. (2017). NF-kB signaling in inflammation. Signal Transduct. Target. Ther..

[51] Ahn J.K., Hwang J.W., Bae E.K., Lee J., Jeon C.H., Koh E.M., Cha H.S. (2012). The role of Raf kinase inhibitor protein in rheumatoid fibroblast-like synoviocytes invasiveness and cytokine and matrix metalloproteinase expression. Inflammation.

[52] Sisto M., Lisi S., D’Amore M., Lofrumento D.D. (2014). Rituximab-mediated Raf kinase inhibitor protein induction modulates NF-kappaB in Sjogren syndrome. Immunology.

[53] Huang Q., Bai F., Nie J., Lu S., Lu C., Zhu X., Zhuo L., Lin X. (2017). Didymin ameliorates hepatic injury through inhibition of MAPK and NF-kappaB pathways by up-regulating RKIP expression. Int. Immunopharmacol..

[54] Li L., Zhao Y.W., Zeng J.S., Fan F., Wang X., Zhou Y., Zhu Z. (2013). Rituximab regulates the expression of the Raf kinase inhibitor protein via NF-kappaB in renal tissue of rats with diabetic nephropathy. Genet. Mol. Res. GMR.

[55] Liu L., Sun Q., Bao R., Roth M., Zhong B., Lan X., Tian J., He Q., Li D., Sun J. (2016). Specific regulation of PRMT1 expression by PIAS1 and RKIP in BEAS-2B epithelia cells and HFL-1 fibroblasts in lung inflammation. Sci. Rep..

[56] Lin W., Wang N., Zhou K., Su F., Jiang Y., Shou J., Liu H., Ma C., Qian Y., Wang K. (2018). RKIP mediates autoimmune inflammation by positively regulating IL-17R signaling. EMBO Rep..

[57] Lin W., Su F., Gautam R., Wang N., Zhang Y., Wang X. (2018). Raf kinase inhibitor protein negatively regulates FcepsilonRI-mediated mast cell activation and allergic response. Proc. Natl. Acad. Sci. USA.

[58] Gao Y., Zhong J., Jiang L. (2018). Raf kinase inhibitor protein protects microglial cells against 1-methyl-4-phenylpyridinium-induced neuroinflammation in vitro. Exp. Cell Res..

[59] Wang J., Du J., Miao C., Lian H. (2016). Raf-kinase inhibitor protein attenuates microglia inflammation in an in vitro model of intracerebral hemorrhage. Cell Mol. Biol..

[60] Wright K.T., Vella A.T. (2013). RKIP contributes to IFN-gamma synthesis by CD8+ T cells after serial TCR triggering in systemic inflammatory response syndrome. J. Immunol..

[61] Gu M., Liu Z., Lai R., Liu S., Lin W., Ouyang C., Ye S., Huang H., Wang X. (2016). RKIP and TBK1 form a positive feedback loop to promote type I interferon production in innate immunity. Embo. J..

[62] Lai R., Gu M., Jiang W., Lin W., Xu P., Liu Z., Huang H., An H., Wang X. (2017). Raf Kinase Inhibitor Protein Preferentially Promotes TLR3-Triggered Signaling and Inflammation. J. Immunol..

[63] Arthur S., Sundaram U. (2014). Protein kinase C-mediated phosphorylation of RKIP regulates inhibition of Na-alanine cotransport by leukotriene D(4) in intestinal epithelial cells. Am. J. Physiol. Cell Physiol..

[64] Lin W., Ma C., Su F., Jiang Y., Lai R., Zhang T., Sun K., Fan L., Cai Z., Li Z. (2017). Raf kinase inhibitor protein mediates intestinal epithelial cell apoptosis and promotes IBDs in humans and mice. Gut.

[65] Beshir A.B., Argueta C.E., Menikarachchi L.C., Gascon J.A., Fenteany G. (2011). Locostatin Disrupts Association of Raf Kinase Inhibitor Protein with Binding Proteins by Modifying a Conserved Histidine Residue in the Ligand-Binding Pocket. Forum Immunopathol. Dis. Ther..

[66] Coussens L.M., Werb Z. (2002). Inflammation and cancer. Nature.

[67] Liu X., Bao Y., Meng W., Yang P., An Y., Ma J., Tang Y., Liu Z., Lu Y., Zhou J. (2019). TROY interacts with RKIP to promote glioma development. Oncogene.

[68] Cook L.M., Hurst D.R., Welch D.R. (2011). Metastasis suppressors and the tumor microenvironment. Semin. Cancer Biol..

[69] Noy R., Pollard J.W. (2014). Tumor-associated macrophages: From mechanisms to therapy. Immunity.

[70] Plaks V., Kong N., Werb Z. (2015). The cancer stem cell niche: How essential is the niche in regulating stemness of tumor cells?. Cell Stem Cell.

[71] Lee S., Wottrich S., Bonavida B. (2017). Crosstalks between Raf-kinase inhibitor protein and cancer stem cell transcription factors (Oct4, KLF4, Sox2, Nanog). Tumour Biol..

[72] Yang S.F., Ma R., Pan L.L., Cao J., Sheng N. (2018). RKIP and peroxiredoxin 2 expression predicts the proliferative potential of gastric cancer stem cells. Oncol. Lett..

[73] Bainer R., Frankenberger C., Rabe D., An G., Gilad Y., Rosner M.R. (2016). Gene expression in local stroma reflects breast tumor states and predicts patient outcome. Sci Rep..

[74] Buschow S.I., Ramazzotti M., Reinieren-Beeren I.M.J., Heinzerling L.M., Westdorp H., Stefanini I., Beltrame L., Hato S.V., Ellebaek E., Gross S. (2017). Survival of metastatic melanoma patients after dendritic cell vaccination correlates with expression of leukocyte phosphatidylethanolamine-binding protein 1/Raf kinase inhibitory protein. Oncotarget.

[75] Wei H., Gao H.Q., Li H.B., Qi S.J., Liu W.L., Xu L., Li H., Liu J.X., Dong Z.M. (2015). Correlation among RKIP expression, NF-kappaB p65 levels, and T-lymphocyte subsets in gastric cardia adenocarcinoma. Genet. Mol. Res..

[76] Crassini K., Pyke T., Shen Y., Stevenson W.S., Christopherson R.I., Mulligan S.P., Best O.G. (2018). Inhibition of the Raf-1 kinase inhibitory protein (RKIP) by locostatin induces cell death and reduces the CXCR4-mediated migration of chronic lymphocytic leukemia cells. Leuk. Lymphoma.

[77] Baritaki S., Huerta-Yepez S., Sakai T., Spandidos D.A., Bonavida B. (2007). Chemotherapeutic drugs sensitize cancer cells to TRAIL-mediated apoptosis: Up-regulation of DR5 and inhibition of Yin Yang 1. Mol. Cancer Ther..

[78] Baritaki S., Suzuki E., Umezawa K., Spandidos D.A., Berenson J., Daniels T.R., Penichet M.L., Jazirehi A.R., Palladino M., Bonavida B. (2008). Inhibition of Yin Yang 1-dependent repressor activity of DR5 transcription and expression by the novel proteasome inhibitor NPI-0052 contributes to its TRAIL-enhanced apoptosis in cancer cells. J. Immunol..

[79] Chatterjee D., Bai Y., Wang Z., Beach S., Mott S., Roy R., Braastad C., Sun Y., Mukhopadhyay A., Aggarwal B.B. (2004). RKIP sensitizes prostate and breast cancer cells to drug-induced apoptosis. J. Biol. Chem..

[80] Baritaki S., Yeung K., Palladino M., Berenson J., Bonavida B. (2009). Pivotal roles of snail inhibition and RKIP induction by the proteasome inhibitor NPI-0052 in tumor cell chemoimmunosensitization. Cancer Res..

[81] Soderstrom T.S., Poukkula M., Holmstrom T.H., Heiskanen K.M., Eriksson J.E. (2002). Mitogen-activated protein kinase/extracellular signal-regulated kinase signaling in activated T cells abrogates TRAIL-induced apoptosis upstream of the mitochondrial amplification loop and caspase-8. J. Immunol..

[82] Baritaki S., Militello L., Malaponte G., Spandidos D.A., Salcedo M., Bonavida B. (2011). The anti-CD20 mAb LFB-R603 interrupts the dysregulated NF-kappaB/Snail/RKIP/PTEN resistance loop in B-NHL cells: Role in sensitization to TRAIL apoptosis. Int. J. Oncol..

[83] Jazirehi A.R., Vega M.I., Chatterjee D., Goodglick L., Bonavida B. (2004). Inhibition of the Raf-MEK1/2-ERK1/2 signaling pathway, Bcl-xL down-regulation, and chemosensitization of non-Hodgkin’s lymphoma B cells by Rituximab. Cancer Res..

[84] Jazirehi A.R., Huerta-Yepez S., Cheng G., Bonavida B. (2005). Rituximab (chimeric anti-CD20 monoclonal antibody) inhibits the constitutive nuclear factor-{kappa}B signaling pathway in non-Hodgkin’s lymphoma B-cell lines: Role in sensitization to chemotherapeutic drug-induced apoptosis. Cancer Res..

[85] Vega M.I., Martinez-Paniagua M., Huerta-Yepez S., Gonzalez-Bonilla C., Uematsu N., Bonavida B. (2009). Dysregulation of the cell survival/anti-apoptotic NF-kappaB pathway by the novel humanized BM-ca anti-CD20 mAb: Implication in chemosensitization. Int. J. Oncol..

